# The relationship between abnormal Core binding factor-β expression in human cartilage and osteoarthritis

**DOI:** 10.1186/s12891-021-04043-9

**Published:** 2021-02-11

**Authors:** Guangdi Li, Mi Zhang, Yuan Huang, Jiafei Yang, Lianghong Dong, Hao Shi, Long Li, Riguang Liu, Jiangwei Li

**Affiliations:** Department of Orthopaedics, The Affiliated Hospital of Guizhou Medical University, Guiyang, China

**Keywords:** Core binding factor-β, Osteoarthritis, MMP-13, IL-1beta, COMP

## Abstract

**Background:**

This study aimed to investigate the effect of abnormal Core binding factor-β expression on proliferation, differentiation and apoptosis of chondrocytes, and elucidate the relationship between Core binding factor-β and osteoarthritis-related markers and degenerative joint disease.

**Methods:**

Cartilage tissues, from healthy subjects and patients with osteoarthritis, were collected for histology and expression of Core binding factor-β, MMP-13, IL-1β, COMP, and YKL-40. Human articular chondrocytes were cultured in vitro, and a viral vector was constructed to regulate cellular Core binding factor-β expression. Cellular proliferation and apoptosis were observed, and osteoarthritis-related inflammatory factor expression and cartilage metabolite synthesis assayed.

**Results:**

Human osteoarthritis lesions had disordered cartilage structure and cellular arrangement, and increased emptying of cartilage lacunae. Normal cell counts were significantly reduced, cartilage extracellular matrix was obviously damaged, and type II collagen expression was significantly decreased. Core binding factor-β was highly expressed in the osteoarthritis cartilage (*p* < 0.001), and MMP-13, IL-1β, COMP and YKL-40 expression were greater than found in normal cartilage (*p* < 0.001). Cellular proliferation in the Core binding factor-β high-expression group was reduced and the total apoptosis rate was increased (*p* < 0.05), while the opposite was found in the Core binding factor-β inhibition group (*p* < 0.01). Compared with normal chondrocytes, high Core binding factor-β expression (Osteoarthritis and CBFB/pCDH groups) was associated with significantly increased MMP13, IL-1β, COMP and YKL-40 protein expression (*p* < 0.01), while Core binding factor-β inhibition (CBFB/pLKO.1 group) was associated with significantly decreased COMP, MMP13, IL-1β and YKL-40 expression in osteoarthritis cells (*p* < 0.001).

**Conclusions:**

Abnormal Core binding factor-β expression might play an upstream regulatory role in mediating abnormal chondrocyte apoptosis and the inflammatory response. On inhibiting Core binding factor-β expression, a delay in cartilage degeneration was expected.

**Trial registration:**

The study was registered for clinical trials in ChiCTR: ChiCTR1800017066 (Reg. Date-2018/7/10).

## Background

Osteoarthritis (OA) is a degenerative disease of the bone and joints, seriously affecting the quality of life of patients [1]. It often occurs in middle-aged and elderly people leading to a high disability rate. Indeed, on a global scale, there were about 303.1 million prevalent cases of hip and knee OA, with an age-standardised prevalence estimate of 3754.2 per 100,000 [2]., bringing huge economic burdens to families of affected patients and society.

OA is characterized by degeneration of the articular cartilage and secondary bone hyperplasia. The pathogenesis of joint diseases caused by mechanical, metabolic, inflammatory and immune factors remains unclear [3]. OA presentation often occurs in the knee, hip, ankle and vertebral joints with clinical manifestations characterized by slow development of joint pain, stiffness, swelling, movement limitation and deformity [4]. The incidence of cardiovascular events and all-cause mortality increase with disease progression [5].

OA is classified into primary and secondary OA. The appearance of primary OA mostly occurs in the middle-aged and elderly, without clear induction, which is related to genetic, obesity and physical factors [6]. The appearance of secondary OA occurs in young adults, secondary to trauma, inflammation, accumulated strain or congenital diseases, and so on [7]. Currently, OA-specific biomarkers are still lacking in the clinical setting. Commonly used drugs for OA treatment include paracetamol, non-steroidal anti-inflammatory drugs (NSAIDs), and opioids. However, NSAID and opioids display a wide variety of marked side effects, so they are not suitable for all patients. Although drugs such as corticosteroids are also commonly used in the clinic, they usually have only short-term efficacy [8]. Therefore, it is of great significance to explore the pathological mechanism of OA in an attempt to discover new targets for the diagnosis and treatment of OA.

Recently, studies have found that Core binding factor-β (Cbfβ) participates in chondrocyte proliferation, differentiation and osteogenesis [9]. The core binding factors are composed of the Cbfα and Cbfβ subunits: the α-subunit is namely a Runx family (Runx) protein (including Runx1–3). Cbfβ does not directly bind DNA, but enhances the binding of Runx to DNA [10]. Cbfβ forms a complex with Runx to regulate the transcription of downstream genes, as well as the maturation and differentiation of hematopoietic stem cells [11]. The binding with Runx2 participates in the differentiation of chondrocytes and subchondral osteocytes, further affecting the development of the bone and joint [12]. Johnson et al. [13] demonstrated that target intervention to disrupt the interaction between the filament protein A and Cbfβ affects the gene transcription of Cbfβ-Runx1, regulates the process of induced differentiation of chondrocytes, and may promote the repair of degenerated cartilage seen in OA. The study suggested that taking Cbfβ and Cbfβ/Runx as putative targets might help in the elucidation of a novel pathway for the early clinical diagnosis and treatment of OA. However, most of the current studies on Cbfβ have investigated osteogenesis and chondrogenesis, and whether the abnormal expression of Cbfβ has an impact on the degenerative disease of the articular cartilage remains unclear.

On the basis of clinical investigation, this study aims to further investigation and discuss the effect of abnormal expression of Cbfβ on proliferation, differentiation and apoptosis of chondrocytes, as well as determining its association with articular cartilage degeneration, with the over-arching aim of providing newly discovered ideas for exploring novel target for the diagnosis, treatment and pathogenesis of OA.

## Methods

### General information

Patients with knee osteoarthritis (KOA) undergoing total knee arthroplasty in the Orthopedic Department of the Affiliated Hospital of Guizhou Medical University from July 2018 to March 2019 were enrolled to the experimental group, and those with traumatic emergency lower limb amputation were enrolled to the control group. The diagnostic criteria for KOA referred to the Guidelines for the Diagnosis and Treatment of Osteoarthritis (2018) and the AAOS Guidelines for Evidence-Based Medicine of Knee Osteoarthritis (2013) of the Orthopedic Branch of the Chinese Medical Association. The study was approved by the Ethics Committee of the Affiliated Hospital of Guizhou Medical University (Approval No. 2018 Lunshen (15)) and registered for clinical trials in ChiCTR (http://www.chictr.org.cn): ChiCTR1800017066. The principle of voluntary participation was adopted for the collection of experimental specimens. Before collection, the subjects or family members were briefed on the study aims and rationale, and this explanation provided them an understanding of the study prior to them signing the consent document. Inclusion and exclusion criteria for study subject enrollment were as follows:

Experimental group: *Inclusion criteria* included the following: (1) met the diagnostic criteria for KOA in this study; (2) no history of knee joint infection or surgical trauma; (3) no history of joint cavity puncture or injection therapy; (4) no history of hormonal, immunosuppressant or other drug therapy; (5) no serious systemic diseases or obvious abnormalities in liver and kidney function. *Exclusion criteria*: (1) chronic consumptive diseases, such as tumors and tuberculosis; (2) other types of arthrosis diseases, such as gout and rheumatoid arthritis; (3) knee deformity and dysfunction caused by congenital factors; (4) patients with psychiatric diseases that were incapable of cooperating with investigators.

Control group: *Inclusion criteria*: (1) older than 18 years of age; (2) no symptoms such as chronic knee pain, no history of knee joint disease, trauma or surgery; (3) no history of hormonal, immunosuppressant or other drug therapy; (4) no history of serious systemic diseases or significant abnormalities in liver and kidney function. *Exclusion criteria*: (1) chronic consumptive diseases, such as tumor and tuberculosis; (2) special conditions such as psychiatric diseases that were incapable of cooperating with investigators; and (3) women during menstruation and pregnancy.

### Experimental procedure

#### Collection and observation of human cartilage tissue

##### Specimen collection

The full cartilage of the femoral condyle and tibial plateau was taken and cut into pieces on a super-clean bench for immediate primary chondrocyte culture. The remaining cartilage tissue was sub-packaged into 5 ml EP tubes: one was immersed and fixed in 4% paraformaldehyde (Cat. No. P1110, Solarbio Science and Technology Co., Ltd., Beijing, China), and the remainder was frozen at − 80 °C.

##### Histological observation of cartilage specimens

HE staining (Hematoxylin and Eosin staining kit, Cat. No. G1120, Solarbio Science and Technology Co., Ltd., Beijing, China): cartilage tissues were fixed in 4% paraformaldehyde for 48 h and decalcified with decalcification solution (G1105, Servicebio Technology Co., Ltd., Wuhan, China) for 4 wks, followed by routine paraffin-embedded sectioning, hematoxylin staining for 10 min, and rinsing with running water. Then, the sections were differentiated for 30 s, immersed in water for 15 min; stained with eosin for 1 min, rinsed and immersed with running water for 2 min, followed by dehydration, clearing, and sealing with neutral gum (Cat. No. G8590, Solarbio Science and Technology Co., Ltd., Beijing, China). The sections were observed under a standard light microscope (BX63, OLYMPUS, Japan).

Safranin O-solid green staining (modified Safranin O-solid green cartilage staining kit, Cat. No. G1371, Solarbio Science and Technology Co., Ltd., Beijing, China): the cartilage tissues were decalcified with EDTA decalcification solution (Cat. No. DD0002, Leagene Biotech Co., Ltd., Beijing, China), embedded in paraffin, sectioned, stained with safranin O-solid green, and observed under a standard light microscope (BX63, OLYMPUS, Japan).

Immunohistochemical staining of collagen type II (COL2A1 polyclonal antibody, Cat. No. bs-10589R, Bioss, Beijing, China) was performed according to the instructions of the ready-to-use immunohistochemical Elivision™ super kit (Elivision™ super HRP Mouse/Rabbit IHC Kit, Cat. No. KIT-9922, Maixin Biotech. Co. Ltd., Fuzhou, China): the cartilage tissues were decalcified with EDTA decalcification solution, embedded in paraffin, sectioned, stained by type II collagen immunohistochemistry, and observed under a standard light microscope (BX63, OLYMPUS, Japan). The integrated optical density (IOD) of positive cells was analyzed by Image Pro-Plus 6.0 software.

##### Western immunoblot

Western immunoblotting was used to detect the expression differences of Cbfβ, MMP13, IL-1β, YKL-40, and COMP in both normal and OA cartilage tissues: protein was extracted from the cartilage tissue in liquid nitrogen, and the protein concentration was determined by the BCA protein assay kit (Cat. No. PC0020, Solarbio Science and Technology Co., Ltd., Beijing, China). Using the measured protein content as reference, equal amounts of protein were added to each well, and then the protein content was verified according to the reference of β-actin (Cat. No. AC026, Abclonal Biotech Co., Ltd., Beijing, China). The experiment was repeated to adjust the amount of protein loading to make consistent comparisons between samples, which were then loaded at 40 μg for each protein for SDS-PAGE, followed by transmembrane blotting, and incubation with the primary antibody, and the secondary antibody respectively (HRP Goat Anti-Rabbit IgG, 1:100, Cat. No. AS014, Abclonal Biotech Co., Ltd., Beijing, China). The membrane was developed by the Bio-Rad chemiluminescent imaging system, and Image J 2X software (https://sourceforge.net/projects/ij2x/) that was used for semi-quantitative analysis of the bands to calculate relative expression. Cbfβ (Cat. No. bs-6984R), MMP13 (Cat.No.bs-10250R), IL-1β (Cat.No.bs-20448R), YKL-40 (Cat.No.bs-10215R), and COMP (Cat.No.bs-10286R) in the cartilage tissues were measured (above antibodies were all purchased from Bioss Biotechnology Co., Ltd., Beijing, China).

#### Human chondrocyte culture

Fresh articular cartilages were cut into pieces and washed in PBS three times, cut to 1 mm^3^, washed twice in PBS, digested with 0.25% trypsin for 30 min, centrifuged at 1500 rpm for 5 min to discard the supernatant, washed twice in PBS, digested with 0.2% collagenase type II in a 37 °C water bath for 3–5 h until the tissue blocks basically disappeared and the digestive fluid became turbid, and then filtered with 150 mesh cell sieve to collect the filtrate at 1500 rpm for 10 min. The supernatant was resuspended in PBS, centrifuged again and then resuspended in 20% FBS high-sugar DMEM culture medium, and then centrifuged at 1500 rpm for 5 min to discard the supernatant, which was repeated twice. Finally, the cells were added to a high-sugar DMEM medium containing 20% FBS and 1% 100x penicillin/streptomycin antibiotics to adjust the density to 0.5 × 10^6^–1 × 10^6^ cells/ml, and cultured at 37 °C, in a 5% CO_2_ in air atmosphere. When the adherent cells were fully confluent at the base of the culture flask, the cells were digested by 0.25% trypsin and subcultured for future use. The third generation cells were selected for subsequent experiments. Normal and OA chondrocytes were cultured respectively. Immunocytochemical staining of type II collagen showed that the cells in both groups were positive with a brown-yellow cytoplasm, and the nucleus was basically unstained, which indicated that the cells were successfully cultured with cartilage characteristics. At the same time, combined with the sampling site, the above cultured cells were confirmed to have met the needs of subsequent studies. When normal chondrocytes were passaged to the 5th cycle or OA chondrocytes to the 4th cycle of culturing, the cells lost their original morphology and had transformed into a fibroblast-like shape. Thus, the third generation of cells was used in the following studies.

#### Construction of over-expression and inhibition of human chondrocyte Cbfβ gene

##### Construction of human CBFB/pCDH lentiviral vector

PCDH cDNA Cloning and Expression Lentivectors, pCDH-MCS-T2A-copGFP-MSCV (Catalog #: cd523a-1, System Biosciences, USA) was used to construct and obtain the Cbfβ gene-overexpressed lentiviral vector plasmid. The complete CDS sequence of the human Cbfβ sequence was searched on GenBank (NCBI Reference Sequence: NM_022845.2; *Homo sapiens* core-binding factor beta subunit (CBFB), transcript variant 1, mRNA; full length of 564 bp). The primer was designed as follows: EcoRI-hCBFB-F: CGG AAT TCA TGC CGC GCG TCG TGC CCG A; Not I-hCBFB-R: TTG CGG CCG CTT AAC GAA GTT TGA GGT CAT. PCR amplification of gene fragments: high fidelity Taq 50 μL PCR: double distilled water 30.5 μL; upstream primer 2 μL; downstream primer 2 μL; template CDNA 2 μL; dNTP 4 μL; Mgcl2 4 μL; 10 EXTaq Buffer (Mg^2+^ free) 5 μL; and EXTaq 0.5 μL in a total of 50 μL in the PCR device. The program was as follows: ① 94 °C, 3 min; ② 94 °C, 30 s; ③ 52 °C, 30 s; and ④72 °C, 1 min; Go to Step 2, total 30 cycles; ⑤ 72 °C, 10 min; Hold at 4 °C. After gene fragment amplification, the Cbfβ gene gel was recovered, and gene fragments and plasmid vectors were annealed. The operation was performed following the instructions found in the pCDH cDNA Cloning and Expression Lentivectors User Manual.

Double enzymatic digestion was comprised of the following: pCDH vector 3 μl; restriction endonuclease EcoRI 1 μl, NotI 1 μl; 10 × Buffer 2 μl and components composed to a volume of 20 μL in ddH2O. Target gene 5 μl; restriction enzyme EcoRI 1 μl, NotI 1 μl; 10 x Buffer 2 μl; and components composed to a volume of 20 μL in ddH2O. The samples were mixed well and digested at 37 °C for 30 min. Expression vector connection: target gene 1 μl; PCDH-GFP 1 μl; 10 x T4 Buffer 1 μl; and T4 DNA ligase 1 μl, following which, the volume was constituted to 10 μL in ddH2O, and incubated at 16 °C overnight.

The ligation product was transformed into *Escherichia coli* DH5alpha. Monoclonal Expansion Culture: the monoclone was selected and inoculated into 5 ml liquid LB culture containing 100 mg/ml Amp resistance selection media, and shaken overnight at 37 °C. The bacterial liquid was transferred into a 7 ml sterilized centrifuge tube and centrifuged at 10,000 rpm for 5 min at room temperature. Plasmids were extracted using the plasmid extraction kits (QIAprep Spin Miniprep Kit, Cat. No. 27104, Qiagen Bio Ltd., Dusseldorf, Germany). The cloned plasmid was used for PCR to identify the insertion of the target gene into the vector; the target gene band (564 bp) was visible after 1% agarose gel electrophoresis of the plasmid targeted gere, and the positive cloned plasmid was identified by sequencing with the sequencing primers of 5′-GGG GTA CAG TGC AGG GGA AAG AAT-3′.

##### Construction of the human CBFB/pLKO.1 lentiviral vector

Using the pLKO.1 - TRC Cloning Vector (Cat. No. Plasmid #10878, Addgene, MA, USA), the Cbfβ-inhibition lentiviral vector plasmid was constructed. shRNA targeting sequences were searched online through http://sirna.wi.mit.edu/, and the human Cbfβ target sequence was found to be: AAG AGA AGC AGG CAA GGT ATA. According to the requirement of the pLKO.1 - TRC Cloning Vector Protocol, the hairpin structure shRNA oligonucleotide was designed and synthesized using a template as follows: human CBFB shRNA: 5’CCGG AAG AGA AGC AGG CAA GGT ATA CTC GAG TAT ACC TTG CCT GCT TCT CTT TTTTTG 3′; 5’AATT CAA AAA AAG AGA AGC AGG CAA GGT ATA CTC GAG TAT ACC TTG CCT GCT TCT CTT 3′, which was completed by following the instructions of the pLKO.1 - TRC Cloning Vector Protocol Version 1.0, and inserting the hairpin-structure shRNA into the pLKO.1 vector.

The DNA oligonucleotide was solubilized in double distilled water to a concentration of 20 uM. The shRNA oligonucleotide template annealing and reaction system was as follows: forward oligo 5 ul; Reverse oligo 5 ul; 10 x NEB buffer-2 at 5 ul; ddH_2_O to 50 ul; 95 °C for 4 min, and cooling to room temperature. The pLKO.1 plasmid double enzyme digestion was as follows: pLKO.1 6 μg; Age I 1 uL; EcoR I 1 uL; 10 x NEB buffer 5 uL; and ddH_2_O to 50 ul with a temperature of 37 °C 1 h. Target fragment of the gel was recovered after 1% agarose gel electrophoresis. The annealed oligos were inserted into the pLKO.1 vector, and the reaction system was as follows: shRNA 2 ul; 10 x NEB T4 DNA ligase buffer 2 ul; pLKO.1 vector 20 ng; NEB T4 DNA ligase 1 ul; and ddH_2_O to 20 ul, which was maintained at 16 °C overnight. After recombination transformation and monoclonal culture, an expanded culture was conducted to extract the plasmid (QIAprep Spin Miniprep Kit, Cat. No. 27104, Qiagen Bio Ltd., Dusseldorf, Germany). The next procedure was cloning plasmid double enzyme digestion to identify the correct insertion of shRNA into the vector: cloned plasmid 1 μg; EcoR I 0.8 uL; Nco I 0.8 uL; 10 × buffer 2 uL; and ddH_2_O to 20 uL; and at a temperature of 37 °C, for 1 h. Finally, 1% agarose gel electrophoresis was conducted on the reaction products, from which two fragments (2 kb and 5 kb) were shown. The positive cloned plasmids were sequenced using the primer: 5′-CAA GGC TGT TAG AGA GAT AAT TGGA-3′.

##### Plasmid transfection

After the plasmids of the human CBFB/pCDH and CBFB/pLKO.1 lentivirus were obtained, the OA chondrocytes were transfected with the plasmids. Cells were digested and counted 1 day before transfection, and cultured in a 6-well plate for transfection at a density of 80%. Mixing ratio of transfection reagent and plasmid mass = 4 μl:2.5 μg, and then 125 μl DMEM was added to each well, as well as 2.5 μg plasmid DNA, and 4 μl Lipo8000™ transfection reagent (Cat. No. C0533, Beyotime Biotech Co., Ltd., Shanghai, China). After premixing in the sterile centrifuge tube, the solution was added to each well, and cultured for 48 h for use.

##### Detection of human chondrocyte proliferation and apoptosis

The chondrocytes were divided into four groups: control group (normal chondrocytes), OA group (OA chondrocytes), CBFB/pCDH group (transfection with CBFB/pCDH), and the CBFB/pLKO.1 group (transfection with CBFB/pLKO.1) according to the previously decribed experiment, followed by experiments of chondrocyte proliferation and apoptosis.

CCK-8 detection: cells in each group were cultured in a 96-well plate for 24 h, and CCK-8 cell counting Kit (cat. No. a311–01, Vazyme Biotechnology Co., Ltd., Nanjing, China) was used for detection. Absorbance values were measured at 450 nm by a microplate reader.

Annexin V/PI flow cytometry apoptosis (CyFlow® Space; SysmexPartec GmbH, Görlitz, Germany): after trypsin digestion and collection of cells in each group by centrifugation, the experiment was conducted following the annexin V-FITC apoptosis detection kit (Cat. No. c1062l, Beyotime Institute of Biotechnology, Shanghai, China) operation manual guidelines.

*Effect of CBFβ gene over-expression and inhibition on the expression of OA-related inflammatory markers and cartilage metabolism markers.*

##### Real-time PCR detection

The total RNA of chondrocytes in each group was extracted and synthesized by cDNA, based on the instruction of the hiscript II Q select RT Supermix for qPCR (+ gDNA wiper) reverse transcription Kit (Cat.no. r233–01, Vazyme Biotechnology Co., Ltd., Nanjing, China). The target gene CDS sequence was searched against the GenBank gene database, and real-time PCR primer sequence as follows:: Human CBFB forward: 5′-TTT GAA GGC TCC CAT GAT TC-3′, reverse: 5′-ATC TTC AAA TTC GCG TGT CC-3′; Human COMP forward: 5′- AGG ACA ACT GCG TGA CTG TG-3′, reverse: 5′- GTG TCC TTT TGG TCG TCG TT-3′; Human MMP13 forward: 5′-TTG AGC TGG ACT CAT TGT CG-3′, reverse: 5′-GGA GCC TCT CAG TCA TGG AG-3′; Human IL-1β forward:5′-GGG CCT CAA GGA AAA GAA TC-3′, reverse: TTC TGC TTG AGA GGT GCT GA-3′; Human YKL-40 forward: 5′-TCA AGA ACA GGA ACC CCA AC-3′, reverse: 5′-AAA TTC GGC CTT CAT TTC CT-3′; Human GAPDH forward: 5′-GAC ATC AAG AAG GTG GTG AAG CAG-3′, reverse: 5′-GTC AAA GGT GGA GGA GTG GGT-3′.

Relative expression was detected by fluorescence quantitative PCR, following the instructions of the AceQ® qPCR SYBR Green qPCR master mix (Cat. No. Q511–01, Vazyme Biotech Co. Ltd., Nanjing, China). The primer was diluted into 5 μM, and mixed with the following agents in order: 10 μl AceQ® qPCR SYBR Green Master Mix, 0.4 μl Forward Primer (5 μM), 0.4 μl Reverse Primer (5 μM), 0.4 μl ROX Reference Dye, Distilled Water (dH2O), which was added to 20 μl, and then amplification on the ABI Prism 7300HT (Real Time PCR amplifier) using two-step PCR. The thermo-cycling conditions constituted of the following: initial denaturation at 95 °C for 10 min (1 cycle); denaturation at 95 °C for 15 s, and annealing/extension for 60 °C for 60 s (40 cycles). DataAssist™ v3.0 Software (ABI) was used to analyze the results based on the 2^-ΔΔCt^ method.

##### Western Immunoblotting

Total protein extraction in each group was conducted as follows: cells were washed three times in PBS, lyzed with 200 ul PMSF RIPA lysate (Cat.No. P0013B, Beyotime Biotech Co., Ltd., Shanghai, China), cooled on an ice-bath for 30 min, and then centrifuged at 12,000 rpm at 4 °C for 5 min. The protein concentration was determined by the BCAassay. The protein supernatant of different groups was collected, and denatured with 5 × SDS loading buffer (20% v/v) and subjected to a boiling water treatment for 10 min. SDS-PAGE was performed at 80 V concentrated gel voltage and 140 V separated gel voltage, followed by PVDF membrane transfer, and then blocking against non-specific antibody binding activity in 5% skimmed milk powder TBS-T solution overnight at 4 °C. The membrane was further incubated with primary antibody diluted with TBS-T containing 5% skimmed milk powder (1: 1000) at 4) (11r (11wder (the detection target was Cbfβ, IL-1β, YKL-40, COMP and MMP13 using β-actin as a reference (all the antibodies used were the same as those described previously above). The membrane was further incubated with a secondary antibody that was diluted in TBS-T containing 5% skimmed milk powder (HRP Goat Anti-Rabbit IgG, 1:5000, Cat.No. AS014, Abclonal Biotech Co., Ltd., Beijing, China) at room temperature for 60 min, and then developed by ECL (WBKLS0100, Millipore, USA), and photographed by Bio-Rad and recorded.

### Statistical method

SPSS version 25.0 software (IBM SPSS statistics 25.0 × 64) was used for statistical analysis. Measurement data were expressed as the mean ± SD. The Student’s t-test was used to compare both groups. One-way ANOVA followed by Tukey’s post hoc test or repeated measures ANOVA was used to determine the significance of the differences between groups. The Chi square test was used for comparisons of clinical count data. An alpha value of *P* < 0.05 was regarded as a statistically significant difference.

## Results

### Study subject inclusion, cartilage acquisition and histological observation

In this study, 53 KOA patients were first selected for total knee arthroplasty, and 22 patients were finally included into the experimental group (obtaining the pathological cartilage tissue) and strictly following the exclusion criteria. There were 9 men and 13 women, with a mean age of 64.09 ± 4.20 years and an average BMI of 25.73 ± 3.84. The K-L grade of the knee joint was determined as being grade IV [14]. According to the inclusion criteria, 8 patients were included in the emergency trauma amputation group as the control group (obtaining normal cartilage tissue), which included 4 men and 4 women, with a mean age of 53.13 ± 13.53 years and a BMI of 22.69 ± 2.71. The K-L grade of the knee joint was 0 [14]. The age (*F* = 13.145, *p* = 0.001) difference for both groups was significantly different (*p* < 0.05), while the BMI showed no difference at all (*F* = 1.40, *p* = 0.247) while the gender composition also showed no different (*χ 2* = 0.197, *p* = 0.698; *P* > 0.05) (Table 1).

**Table 1:** Comparison in general data

	Experimental group	Control group	*p*
Age	64.09 ± 4.20	53.13 ± 13.53	0.001
BMI	25.73 ± 3.84	22.69 ± 2.71	0.247
Gender (male/female)	9/13	4/4	0.698

**Fig. 1:**
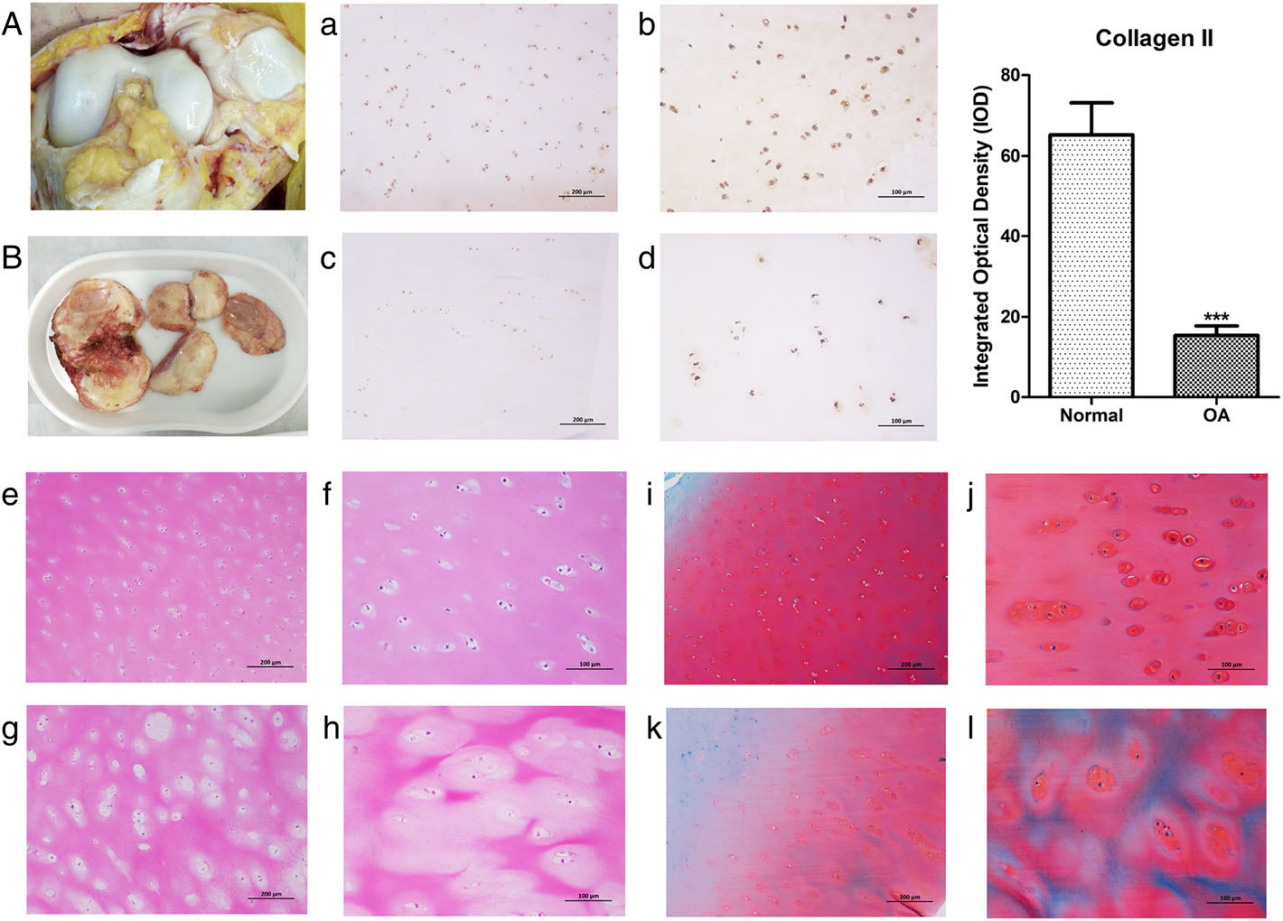
Normal human knee joint cartilage (A) and OA diseased cartilage (B) macroscopic tissue structure; Collagen type II immunohistochemical staining: normal cartilage (a-*100×, scale bar = 200* μm; b-*200×, scale bar = 100 μm*) and OA cartilage (c-*100×, scale bar = 200 μm*; d-*200,×scale bar = 100 μm*); HE staining: normal cartilage (e-*100×, scale bar = 200 μm*; f-*200×, scale bar = 100 μm*) and OA cartilage (g-*100×, scale bar = 200 μm*; h-*200×, scale bar = 100 μm*); Safranin O-solid green staining: normal cartilage (i-*100×, scale bar = 200 μm*; j-*200×, scale bar = 100 μm*) and OA cartilage (k-*100×, scale bar = 200 μm*; l-*200×, scale bar = 100 μm*)

The macroscopic structure of the normal cartilage in the control group (Fig. 1a) and the OA diseased cartilage in the experimental group (Fig. 1b) were significantly different. The collected cartilage of the knee joint in the control group showed a milky white appearance with a smooth and shiny surface but with no evidence of cracks, erosion or ulcer formation. In the experimental group, the specimens showed a yellow and dark color, with uneven surfaces, fissures, thin cartilage, and exposed subchondral bone. By HE staining, safranin O staining and type II collagen immunohistochemical staining, we examined the morphology and appearance of collagen as a key component of the cartilage matrix (mainly as type II collagen). The staining results showed that when compared with normal cartilage (Fig. 1a and b), the expression of the type II collagen protein in the OA cartilage was significantly decreased (*p* < 0.05). It can be seen that the normal phenotype of chondrocytes in the OA diseased cartilage persisted; however, the expression of type II collagen had decreased, and the overall structure of cartilage tissue was disordered. HE staining showed that the nuclei of the normal cartilage (Fig. 1e and f) were dark blue, the cytoplasm and cartilage matrix were pink, and demonstrated uniform staining. The cells on the superficial layer were small and round, and were arranged parallel to the cartilage surface. The cells in the deep layer were larger and oval or round. In the experimental group (Fig. 1g and h), the structure was disordered. There were many empty cartilage lacunae with a disordered cellular arrangement. Chondrocyte necrosis was observed in the superficial and deep layers, and the number was significantly reduced. Safranin O solid green staining showed that the matrix of the normal cartilage (Fig. 1i and j) was uniformly stained red, and the subchondral bone was green, with a clear boundary between the cartilage and subchondral bone. The OA structure of cartilage (Fig. 1k and l) was disordered, the staining of the cartilage matrix was lighter, and the range of red staining was minimal, suggesting that the diseased cartilage matrix was destroyed and reduced.

### Expression of Cbfβ and OA related inflammatory factors and cartilage metabolic markers in OA cartilage tissue

**Fig. 2:**
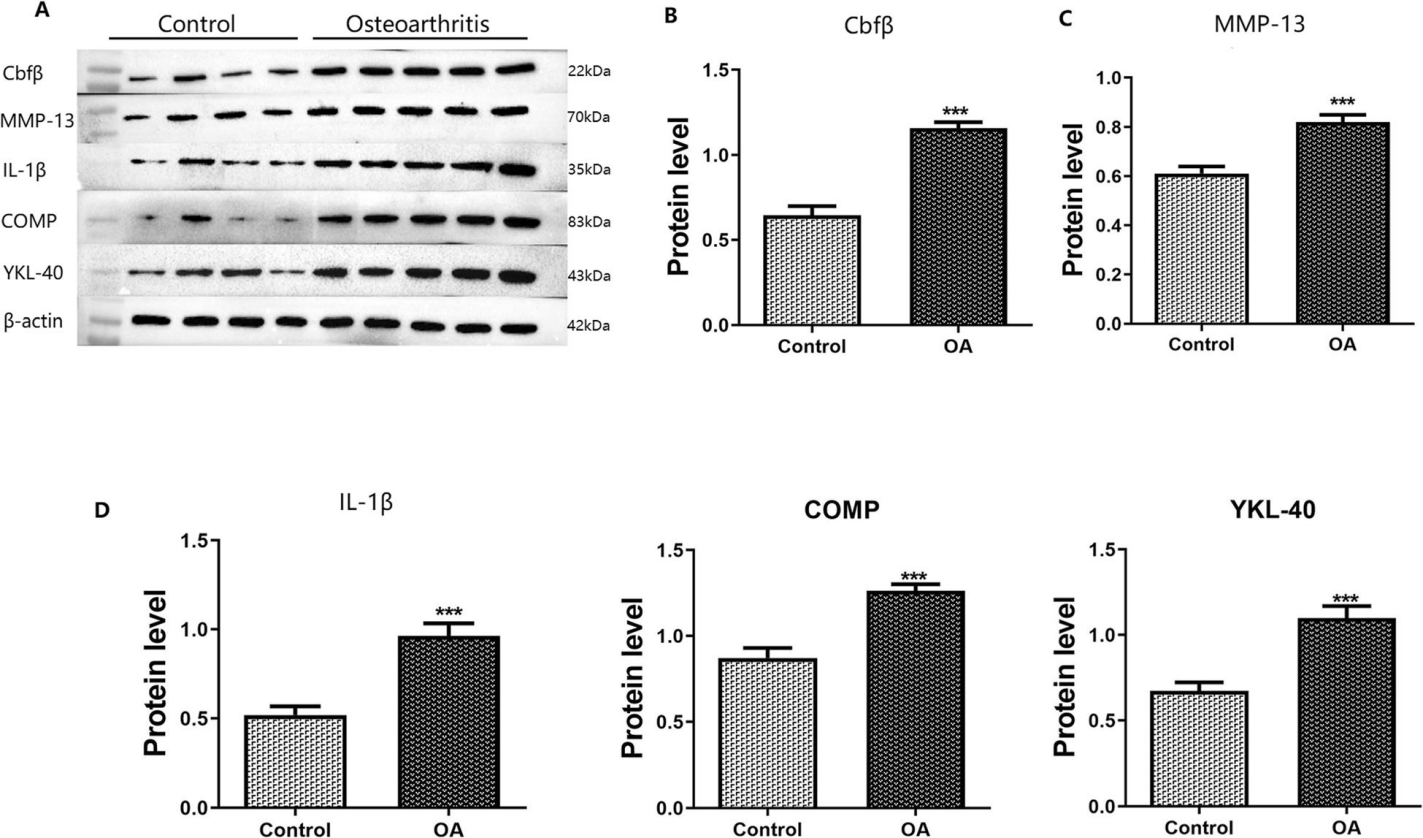
Western blot analysis of human cartilage tissue, A: Western blot band map of each protein, B-F: quantitative histogram of protein bands for the expression of Cbfβ, MMP-13, IL-1β, COMP, and YKL-40 (***: *p* < 0.001)

Cbfβ was highly expressed in human OA diseased cartilage tissues. In addition, the expression of inflammatory factors and cartilage metabolites, including MMP-13, IL-1β, COMP, and YKL-40 that were closely related to OA, were higher than those found in normal cartilage tissues. It was found that the expression of each marker was significantly different when comparing the OA with the control group (*p* < 0.001; Fig. 2).

### Effect of Cbfβ gene over-expression and inhibition on human OA chondrocyte growth

**Fig. 3:**
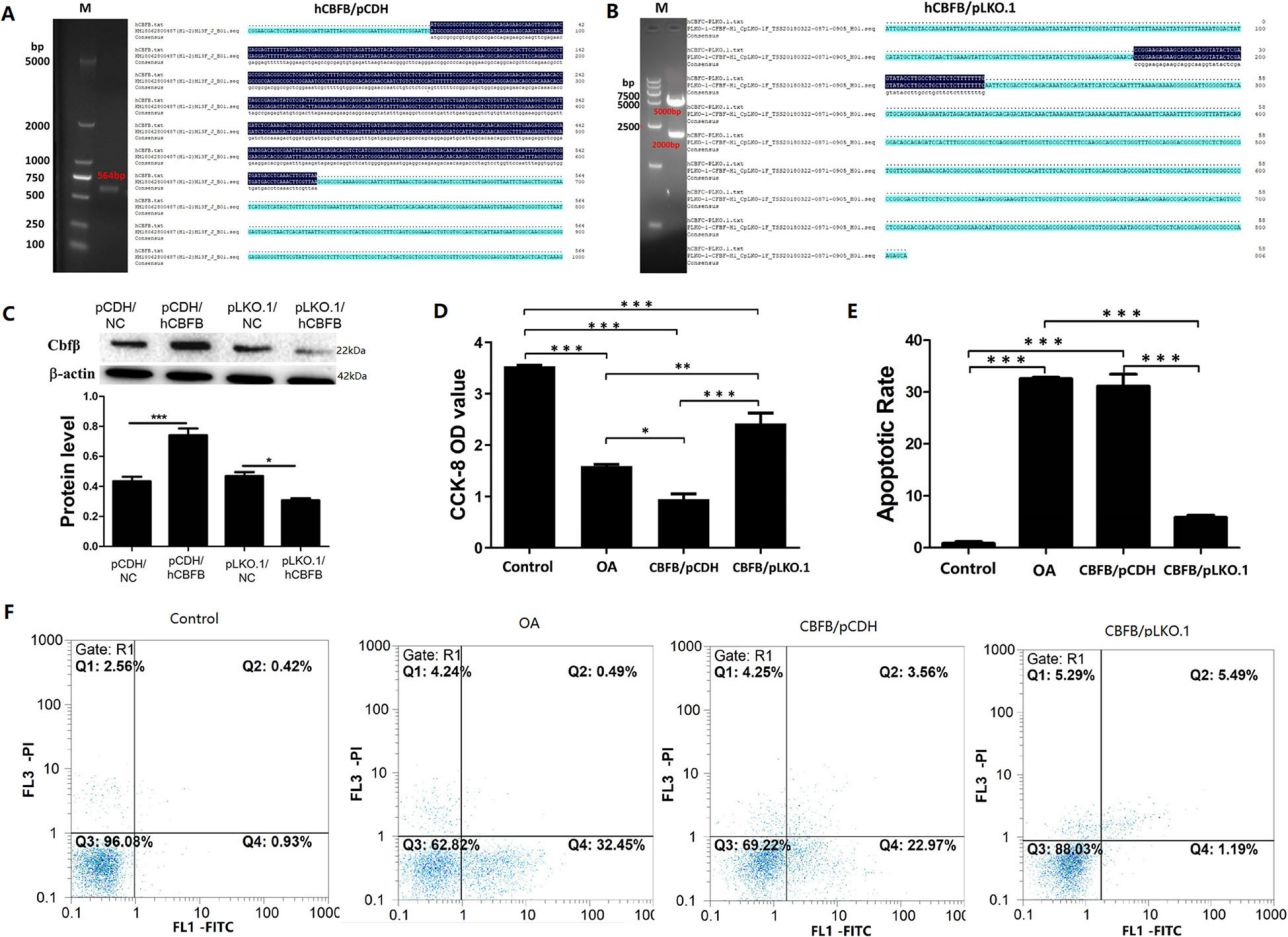
A. Showing the PCR amplification band of the positively cloned CBFB/pCDH plasmid vector was 564 bp, which was consistent with the sequence map. B. CBFB/ plkko.1 plasmid vector positive clones were 5 kbp and 2 kbp after double enzymatic digestion, and the sequencing results were consistent. C. Validating Western immunoblot results of plasmid transfection efficiency; D. cell proliferation in each group as detected by the CCK-8 assay; E, F. cellular apoptosis in each group as detected by Annexin V/PI double staining (*: *p* < 0.05, **: *p* < 0.01, ***: *p* < 0.001)

After sequence identification, the human CBFB/pCDH lentiviral vector (Fig. 3a) and the human CBFB/pLKO.1 lentiviral vector (Fig. 3b) were effectively constructed. Viral plasmids were transfected into OA lesional chondrocytes. It was determined that Cbfβ over-expression and inhibition occurred significantly in both groups (Fig. 3c). The result of CCK-8 analysis (Fig. 3d) of the four groups indicated that abnormal expression of Cbfβ had a significant negative effect on the proliferation and growth of human articular chondrocytes. Compared with the control group, the proliferation of cells in the OA group decreased significantly; moreover, the over-expression of Cbfβ decreased cell viability, and the inhibition of Cbfβ significantly increased cell proliferation. This indicated that the increased expression of Cbfβ inhibited cell proliferation; however, the inhibition of Cbfβ gene expression had an opposing effect. The results of Annexin V/PI flow cytometry apoptosis (Fig. 3e and f) showed that the apoptotic rate (Q2 + Q4) in the OA and CBFB/ pCDH group with an abnormally increased Cbfβexpression was significantly increased as compared the control group (*p* < 0.001), but that of the CBFB/pLKO.1 group was significantly down-regulated (*p* < 0.001), which suggested that Cbfβinhibition effectively reduce early and late apoptosis (Q4), and number of necrotic (Q2) OA chondrocytes.

### Effects of Cbfβ over-expression/inhibition on the expression of OA related inflammatory factors and cartilage metabolic markers

**Fig. 4:**
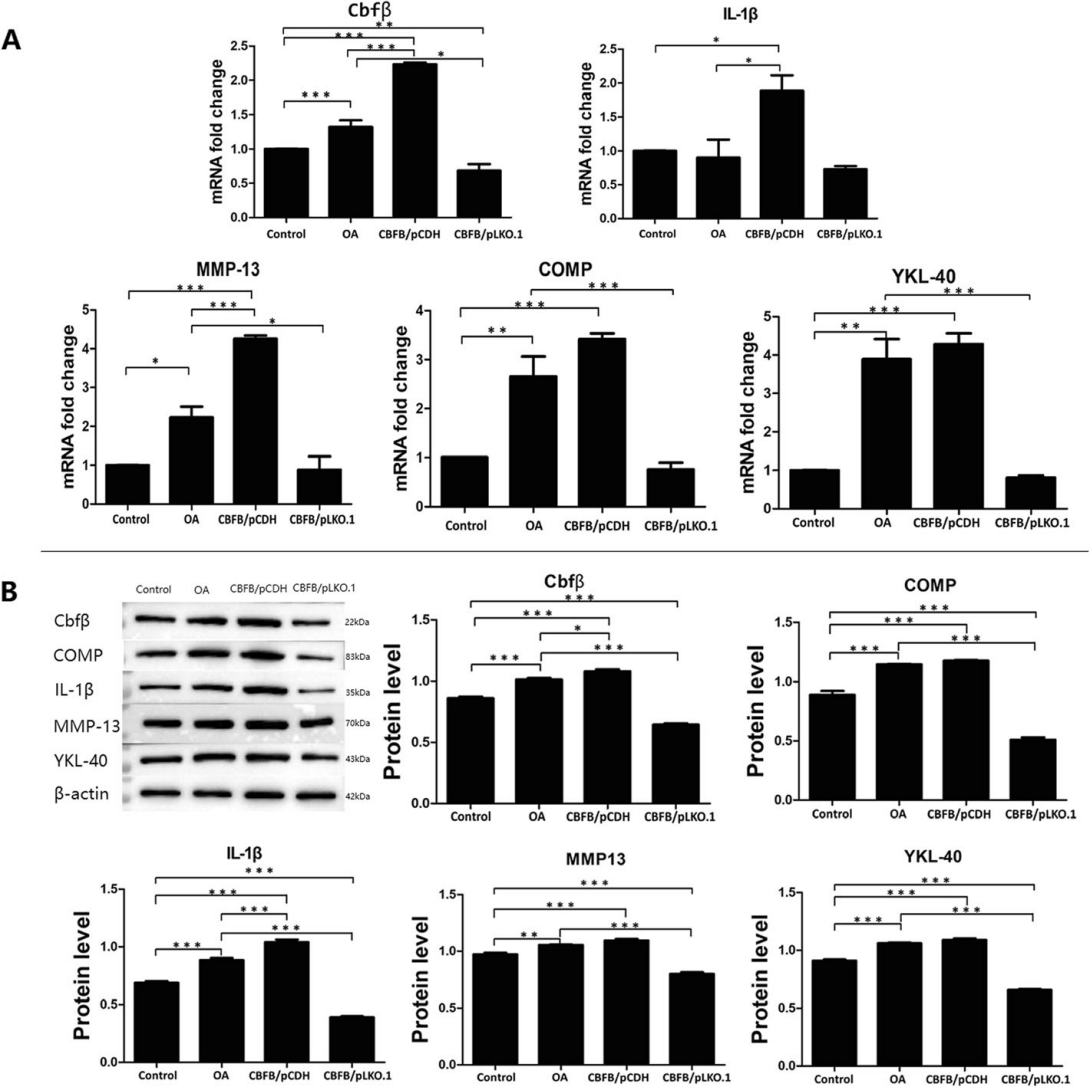
A. Statistical histogram of real-time PCR results, B. Bands and statistical histograms of Western blot results (*p < 0.05, **p < 0.01, ***p < 0.001)

Real-time PCR (Fig. 4a) and Western blot (Fig. 4b) were used to detect inflammation-related factors and marker products (i.e., IL-1beta, MMP-13, COMP, YKL-40) of cartilage metabolic abnormalities closely related to OA in the control group, OA group, CBFB/pCDH group, and the CBFB/pLKO.1 group. Regulating the expression of Cbfβ in chondrocytes induced changes in expression of the four OA markers (IL-1beta, MMP-13, COMP, YKL-40). Real-time PCR results showed that (Fig. 4a) mRNA expression of Cbfβ and other related markers were all increased in the OA group as compared with the control group, and there were significant differences in the observed increases in COMP, MMP13 and YKL-40 (*p* < 0.05). Compared with the OA group, the mRNA expression levels of MMP13 and IL-1β in OA cells were significantly increased when Cbfβ was over-expressed (*p* < 0.05), and the expression of COMP and YKL-40 was somewhat up-regulated. Compared with the OA group, COMP, MMP13 and YKL-40 mRNA expression in chondrocytes was also significantly decreased after inhibition of Cbfβ expression (*p* < 0.05). Western blot results showed that when compared with the control group, protein expression of Cbfβ and other related markers were significantly increased in the OA group (*p* < 0.01). When compared with the OA group, the expression of other cellular markers showed an increasing trend when Cbfβ was over-expressed, and the expression of COMP, MMP13, IL-1β and YKL-40 in chondrocytes was significantly decreased when Cbfβ expression was inhibited (*p* < 0.001).

## Discussion

Joint degenerative diseases, such as osteoarthritis are closely related to the functional changes of chondrocytes in the articular cartilage tissue [15]. Abnormal cell proliferation, differentiation and apoptosis leads to cartilage matrix degeneration and calcification, which indicates that the pathogenesis of osteoarthritis is closely related to the proliferation, differentiation and death of chondrocytes. Studies have confirmed that multiple OA-related changes in cytokines and molecular signals can induce chondrocyte abnormalities [16–19], which indicates that studying the mechanism of chondrocyte degeneration and apoptotic abnormalities are promising approaches to help guide early diagnosis and treatment protocols for osteoarthritis in the clinic. At the same time, development of specific biological and drug preparations for chondrocyte proliferation, differentiation and apoptosis mechanisms is expected to effectively delay the development of the degeneration of the OA articular cartilage [15].

Recently, it has been found that core binding factor β-subunit (Cbfβ) is closely related to chondrocyte differentiation and osteogenesis [9]. Cbfβ controls the balance between chondrocyte proliferation and differentiation, and promotes chondrocyte proliferation and osteoblast differentiation by up-regulating Ihh expression, and inhibits PPR expression to enhance chondrocyte differentiation [20]. Miller et al. [21] established a transgenic model of Cbfβ mice, in which the promoter and enhancer of the Tek gene were used to express the fusion proteins of GFP and Cbfβ in the mice with Cbfβ defects. The mice died due to severe abnormal bone development after birth. Simultaneously, it has been confirmed that [12] mice with abnormal Cbfβ expression died one day after birth. Due to abnormal development of chondrocytes, both intramembranous and endochondral osteogenesis were significantly inhibited. However, the relationship between abnormal expression of Cbfβ and degenerative diseases like OA has not been clearly clarified up to now.

In this study, biomarkers such as OA-related cartilage metabolites and inflammatory factors were detected in the cartilage. The results showed that Cbfβ was highly expressed in human OA diseased cartilage. Meanwhile, the expression of protein markers to include IL-1 β, MMP-13, comp and YKL-40 that were closely related to OA were significantly higher than that seen for normal cartilage.

Cartilage is composed of chondrocytes and extracellular matrix (ECM). Chondrocytes synthesize and secrete matrix components. In the inflammatory condition and during OA progression, significant changes in matrix degradation proteins provokes ECM remodeling. Changes in ECM will affect the chondrocyte microenvironment, which will further promote OA progression under inflammatory condition. Many studies have shown that functional changes in cartilage caused by proliferation, differentiation and apoptosis of chondrocytes are related to the degenerative joint diseases. Abnormal apoptosis often leads to degeneration and calcification of the cartilage matrix [15]. As shown in Fig. 1, when compared with the normal diseased OA cartilage, the color was obviously dull, and the surface was not smooth with many cracks, and the cartilage was inelastic with uneven thickness, and even some joint areas were obviously degenerated and missing. Histological section staining showed that the cartilage structure level was disordered, and displayed more empty cartilage lacunae with a disordered cellular arrangement. In addition, normal cells were decreased significantly in both the surface and deep layer, the destruction of the cartilage ECM was obvious, and the expression of type II collagen in tissues was significantly decreased.

It is widely known that there is a “silent period” in the onset of osteoarthritis, in which patients may not have any clinical symptoms, nor obvious abnormalities by imaging examination. However, cartilage tissues might display extensive metabolic changes [22]. Cartilage destruction and local inflammatory response are key pathological changes seen in OA, and thus biomarkers associated with OA are mainly associated with cartilage metabolites and inflammatory factors [23]. In the process of cartilage degeneration, COMP, YKL-40, KS, CTX-II, PIICP and other small molecular products of cartilage matrix degradation are released gradually, and change with progression of the disease [22]. Verma et al. [24] confirmed that the synthesis and degradation of COMP is closely related to cartilage metabolism. YKL-40 [25] expression levels indirectly indicate joint destruction, which can be used to evaluate the degree of joint degeneration/destruction.

Local inflammatory responses are also one of the most important pathological changes in OA development. Inflammatory mediators break the dynamic balance of cartilage matrix metabolism by affecting the metabolic activity of normal chondrocytes, which accelerates cartilage destruction [26]. The inflammatory markers, especially interleukins [27] (ILs) and matrix metalloproteinases [28–30] (MMPs) have been widely investigated. In previous studies, IL-1β has been shown to play a key role in the development and progression of OA [31, 32]. The results of Özler K et al. [33] showed that MMP-13 expression in the joint fluid of patients with severe KOA was significantly related to the WOMAC score. As a specific biomarker of OA, MMP-13 could be used to determine the extent of cartilage damage to evaluate disease severity.

This is also concordant with the results of our previous detection assays on the pathological cartilage. Combined with the results of previous studies, Cbfβ was selected as the main target, as well as inflammatory related factors and cartilage metabolites including IL-1β, MMP-13, COMP and YKL-40, as the reference for chondrocyte abnormality and degeneration in the later stage of the disease. As a chaperonin of the Runx family of transcription factors, Cbfβ participates in cellular metabolic processes, such as osteogenesis and chondrogenesis, and plays an important role in chondrogenesis [9, 12, 34]. Based on previous conclusions, it was speculated that Cbfβ might play an important role in chondrocyte apoptosis and cartilage tissue degeneration in OA pathogenesis, but the specific role remained to be elucidated.

In this study, a significant abnormal increase in Cbfβ was found in diseased cartilage tissue of OA patients and abnormal changes in various cartilage metabolites and inflammatory factors closely related to OA disease. Thus, it was speculated that abnormally increased expression of Cbfβ in the cartilage tissue of osteoarthritis might play an important regulatory role upstream of the pathogenic pathway. To further clarify the effect, after culturing normal and OA chondrocytes in vitro, we performed Cbfβ gene over-expression and inhibition treatment on OA-diseased chondrocytes by lentiviral transfection. Finally, two phenotypic chondrocytes, CBFB/pCDH (Cbfβ over-expression) and CBFB/pLKO.1 (Cbfβ expression inhibition), were obtained. The results of CCK8 cell proliferation showed that the in vitro proliferation of cells was significantly enhanced after expression of CBFB/pLKO.1 was inhibited (*p* < 0.01). However, after further over-expression of Cbfβ in diseased chondrocytes, the proliferation in the CBFB/pCDH group had further declined. The proliferation of normal chondrocytes was higher from that of the other three groups in CCK8 assays. However, the in vitro cell studies showed that inhibiting and regulating Cbfβ expression in OA diseased cells effectively improved the proliferative activity of chondrocytes. Annexin V/PI flow cytometry also confirmed that after the inhibition of Cbfβ gene expression in the CBFB/pLKO.1 group, the viable cell density was significantly increased from 62.82 to 88.03%, and that of early apoptosis was significantly decreased from 32.45 to 1.19%.

Our study indicated that selective up-regulation of Cbfβ expression in chondrocytes was associated with a significant increase in the total apoptotic rate, and the cell proliferative activity was also reduced. However, inhibition of Cbfβ expression could regulate and reduce the total apoptotic rate, and significantly increase the cell proliferative activity, indicating that Cbfβ might be closely related to pathological manifestations, such as abnormal chondrocyte apoptosis and cartilage tissue degeneration. Indeed, the apoptotic results of the Annexin V/PI flow cytometry assay demonstrated that after over-expression of Cbfβ in the diseased chondrocytes of the CBFB/pCDH group, the viable cell density, the total apoptotic rate and the general expression of chondrocytes in OA lesions were quite similar. While, the count of living cells and the total apoptotic rate in the CBFB/pLKO.1 group had also significantly improvement. Will it also affect the expression of OA-related inflammatory factors and cartilage metabolites in cells? To answer that question, the expression of OA-related inflammatory factors and cartilage metabolites in chondrocytes of each group was analyzed at both the gene and protein levels. The results of real-time PCR and Western blotting showed that there was also a certain expression level of Cbfβ, IL-1β, MMP-13, COMP and YKL-40 in normal chondrocytes of the control group (Fig. 4); however, there was a significant increase in Cbfβ in the OA group (*p* < 0.001). Simultaneously, the expression of the above inflammatory factors and cartilage metabolites were all increased – at least to different extents, which was consistent with our findings.

In the study, we also processed OA chondrocytes by over-expression and inhibition of Cbfβ, which were then divided into the CBFB/pCDH group (Cbfβ over-expression) and the CBFB/pLKO.1 group (Cbfβ expression inhibition). The expression of COMP and YKL-40 in the CBFB/pCDH group showed slight increases in detection by real-time PCR and Western immunoblot analysis. Although there was no significant statistical difference with the OA group (*p* > 0.05), the COMP and YKL-40 expression in the CBFB/pCDH and the OA group were significantly higher than was found in normal cells (*p* < 0.01). Studies have shown that COMP [24] and YKL-40 [25] metabolites were mainly produced by chondrocytes, and were abnormally increased in chondrocyte degeneration or the inflammatory process, which can be used as diagnostic indices for articular cartilage degeneration and destruction.

The above results indicated that an abnormal increase in Cbfβ in chondrocytes significantly affected the apoptosis rate and proliferative activity of cells, and caused an abnormal increase in OA-related cartilage metabolites and inflammatory factors, which was consistent with the characteristics of Annexin V/PI flow cytometry apoptosis assay in this study. In the CBFB/pLKO.1 group, with significantly inhibited Cbfβ expression, the protein expression of disease-related inflammatory factors and cartilage metabolites was significantly decreased as compared with those of the OA group (*p*<0.001). The result of the Annexin V/PI flow cytometry and CCK-8 detection assays in this study indicated that in the pathological mechanism of OA development, Cbfβ played an upstream regulatory role in the inflammatory response and abnormal apoptosis signaling pathway. The abnormal expression of Cbfβ might be a key factor that induces the inflammatory response and abnormal apoptosis of chondrocytes.

Cbfβ plays a regulatory role in the dynamic balance between chondrocyte proliferation and differentiation [20]. As a co-transcription factor of Runx2 [35, 36], Cbfβ forms a heterodimer with the Runx2 protein, playing an important positive regulatory role in chondrocyte differentiation and osteogenesis [37]. Thus, abnormal expression of Cbfβ is closely related to the apoptotic changes of articular chondrocytes and cartilage tissue degeneration. In the study, vector virus was used to regulate Cbfβ in human diseased chondrocytes in vitro, demonstrating a significant effect of Cbfβ regulatory changes on the proliferation, differentiation and apoptosis of chondrocytes. The inhibition of Cbfβ expression significantly reduced OA-related biomarkers to include IL-1β, MMP-13, COMP, and YKL-40. Moreover, the abnormal proliferation and apoptosis of OA-diseased chondrocytes was improved. Thus, the inhibition of Cbfβ expression might play a role in delaying cartilage degeneration, which was expected to provide a novel pathway for the exploration of new targets for the diagnosis, treatment, and pathogenesis of OA.

## Conclusions

In this study, there was a significant abnormal increase in Cbfβ in the articular cartilage of OA patients. Simultaneously, the expression of inflammatory factors and cartilage metabolites closely related to OA was also significantly increased, indicating that the expression changes in Cbfβ was closely related to the occurrence and development of human cartilage degeneration. Our study provides a valuable clinical investigation and laboratory data for the study of OA pathogenesis; however, it has some limitations due to the limited clinical sample size. Future studies will need multi-center clinical trials with large sample sizes. By expanding the sample size of clinical studies and the diversity of included samples, and further connecting with more upstream multi-level regulatory gene expression studies on circRNA, lncRNA, miRNA and other cellular and molecular levels, it will be helpful to clarify the important role of Cbfβ and related markers in osteoarthritis pathogenesis.

## Data Availability

The datasets used and/or analyzed during the current study are available from the corresponding author on reasonable request.

## Abbreviations

OA: Osteoarthritis
NSAIDs: Non-steroidal anti-inflammatory drugs
KOA: Knee osteoarthritis
Cbfβ: Core binding factor β-subunit
ECM: Extracellular matrix
ILs: Interleukins
MMPs: Matrix metalloproteinases
MMP-13: Matrix metalloproteinase-13
